# Association of travel burden with colorectal cancer outcomes in resource-limited settings of Kashmir, India

**DOI:** 10.3332/ecancer.2026.2136

**Published:** 2026-06-02

**Authors:** Saquib Zaffar Banday, Malik Tariq Rasool, Sheikh Zahoor Ahmad, Lekshmi Shenoy, Aaqib Zaffar Banday, Maniza Ayub, Bishal Gyawali

**Affiliations:** 1 Department of Medical Oncology, State Cancer Institute, Sher-i-Kashmir Institute of Medical Sciences, Srinagar, Kashmir 190011, India; 2 Department of Medical-Hematoncology and Stem Cell Transplant, Paras Hospital, Srinagar, Kashmir 190001, India; 3 Department of Radiation Oncology, State Cancer Institute, Sher-i-Kashmir Institute of Medical Sciences, Srinagar, Kashmir 190011, India; 4 Department of Surgical Oncology, State Cancer Institute, Sher-i-Kashmir Institute of Medical Sciences, Srinagar, Kashmir 190011, India; 5 Department of Surgical Oncology, Paras Hospital, Srinagar 190001, India; 6 Department of Pediatrics, Government Medical College, Srinagar, Kashmir 190010, India; 7 Department of Pathology, Sher-i-Kashmir Institute of Medical Sciences, Srinagar, Kashmir 190011, India; 8 Department of Oncology, Queen’s University, Kingston, ON K7L 3N6, Canada; 9 Department of Public Health Sciences, Queen’s University, Kingston, ON K7L 3N6, Canada; 10 Division of Cancer Care and Epidemiology, Queen’s University, Kingston, ON K7L 3N6, Canada

**Keywords:** colorectal cancer, low-middle income countries, resource-limited setting, time toxicity, travel burden

## Abstract

**Background:**

The impact of travel burden, which contributes to both financial and time toxicities, on cancer treatment and outcomes remains largely understudied, especially in resource-limited settings. We assessed the impact of travel burden on the outcomes of patients with colorectal cancer (CRC) treated at a regional cancer center in the resource-limited context of Kashmir, India.

**Methods:**

This was a retrospective study including all patients with newly diagnosed CRC in 2022 at the State Cancer Institute, Sher-i-Kashmir Institute of Medical Sciences, Srinagar, North India. Travel burden was recorded as the average time required to traverse the shortest distance between the cancer center and the place of the patient’s residence. We estimated the travel burden, analyzed the association of travel burden with survival outcomes, and compared outcomes for patients who traveled from their homes for treatment (Group A) to those who rented apartments near the hospital to complete treatment (Group B).

**Results:**

263 patients (42.6% females) with CRC were included, including patients who traveled from their homes (group A) and those who rented apartments near the hospital to complete treatment (group B). Group A patients (N = 178) traveled a median of 22 kms (48 minutes) to reach the cancer center, while Group B patients (N = 85) resided a median of 75 kms (130 minutes) away from the hospital. Travel time did not correlate with symptom duration before diagnosis of CRC.

In group A, patients with the highest travel burden (Q4 travel time) had poorer outcomes than other patients (Q1–Q3 of travel time) (18-month overall survival (OS) of 65% versus 83.6%, adjusted HR 2.5 (95% CI 1.2 to 5.2)). 18-month OS in group B was higher than that for group A patients (85.6% versus 78.9%, p = 0.056).

**Conclusion:**

Our study demonstrates that travel burden is associated with poorer outcomes in patients with CRC.

## Highlights

- Patients with colorectal cancer in Kashmir, India face a substantial travel burden.
- One-third patients had to rent an apartment near the cancer center for treatment.
- Travel time did not correlate with symptom duration.
- Patients with the highest travel burden had a substantially poorer survival.
- Patients who rented an apartment nearby had longer survival than those who traveled to cancer center.

## Introduction

Although the outcomes for patients with colorectal cancer (CRC) have substantially improved globally over the last few decades, the mortality trends in resource-limited settings (RLS) remain disappointing [1, 2]. One of the reasons for these differences in outcomes is probably disparities in access to treatments, including access to comprehensive cancer-care facilities [3]. Although poor access to therapy in RLS is typically attributed to financial toxicity, there is also a component of travel burden that can contribute to poor access to therapy. In high-income countries, time toxicity is being increasingly recognised as an important factor to consider in therapeutic decision-making, including for patients with CRC [4]. This time toxicity is typically quantified in terms of healthcare contact days; however, in RLS, one healthcare contact can encompass several days spent on travel alone, due to centralisation of cancer care and lack of fast and convenient means of transport [5]. Thus, the burden of travel time can be even more substantial for patients treated in RLS.

Multimodality treatment for CRC can entail a substantial time commitment from patients. For patients living in geographically remote locations, reaching a cancer center could mean upwards of many hours of journey one way, and making such a commute frequently can lead to a substantial travel burden and financial toxicity on patients. This can also presumably affect cancer outcomes. However, to our knowledge, travel burden and its effect on CRC outcomes have been under-studied, much less from RLS. Thus, in this study, we aim to study the extent of travel burden (travel time and distance) and its relationship with patient outcomes in patients receiving treatment at a regional cancer center in the RLS of Kashmir, India.

## Methods

This study received ethical approval from and was conducted at the State Cancer Institute (SCI), Sher-i-Kashmir Institute of Medical Sciences (SKIMS), Srinagar, Kashmir, North India. SCI is the region’s only tertiary care health facility that provides comprehensive cancer management facilities. Kashmir is located in the Himalayas with many areas having limited land connectivity, which is especially compromised during the harsh winter. In addition, Kashmir also suffers from political unrest and is frequently an area of conflict and war. Thus, this area represents a unique RLS with geographically remote terrain, poor transport systems and occasional conflicts.

This is a retrospective study using the medical records of patients to confirm their eligibility and capture clinical characteristics, supplemented by telephonic follow-up by the research team between January and July 2025 to confirm their place of residence at the time of receipt of treatment and survival outcomes. All patients with newly diagnosed CRC with documented adenocarcinoma histology and treated at the SCI between 1st January 2022 and 31st December 2022, were enrolled in the study. These dates were selected because this was the first full calendar year of routine cancer care at the institution post-COVID-19 (selected to avoid the confounding effect of travel restrictions and other constraints), and to allow reasonable time for survival events to occur before data analysis. Patients with a previous history of cancer or any form of travel-limiting physical disability were excluded. No funding was received for performing this study.

### Variables studied

Data on patient demographics, such as age, sex and occupation, were captured in the database. Although the address is typically captured in the database, we know from personal experience that many patients rent an apartment or stay at a hotel nearby during treatment, and this information is not captured. Therefore, we called every patient to confirm this data. Informed verbal consent was obtained from each patient for this study. Accordingly, we grouped our patients into two categories – Group A consisted of patients who traveled from their homes for treatment, and Group B constituted patients who rented apartments/accommodations near the cancer center to complete treatment. In addition, clinical characteristics such as histology, pathological features, treatment offered, treatment received and outcomes were recorded from medical records. Overall survival (OS) was defined as the time from initiation of treatment to death from any cause or last follow-up. Patients alive at the time of analysis were censored. Survival status was also confirmed during the telephone follow-up.

Travel burden was quantified in terms of travel time and travel distance, which was calculated as the shortest time required to traverse the shortest distance between SCI SKIMS and the place of the patient’s residence while receiving chemotherapy. This was both asked of the patient during telephone follow-up and verified using Google Maps, and the shortest time was recorded. This use of Google Maps to measure travel burden is consistent with previous studies in this space [6].

### Statistical analysis

The magnitude of travel burden is described using descriptive statistics wherein continuous variables have been expressed as median (Q1, Q3). Relationship with travel time was studied for symptom duration (time from onset of symptoms to diagnosis), advanced stage at presentation and time to treatment initiation (time from first consultation for cancer to initiation of treatment). These analyses were conducted using Spearman’s rank correlation analysis and Mann-Whitney *U* or Kruskal-Wallis tests.

For patients in Group A, who commute from home to the cancer center, the travel burden was categorised into high versus low. Patients in the highest quartile (Q4) of travel time were categorised as high travel burden, and the other three quartiles (Q1–Q3) were considered as having low travel burden. We made this distinction to specifically assess the impact of travel burden in patients who need to commute the most for cancer care. For Group B patients, because they started living near the hospital, this categorisation was not done.

The Log Rank test was used to compare survival differences between variables. Hazard ratios were calculated using the Cox proportional hazards model. Independent predictors of outcome were determined using stepwise Cox regression analysis. Variables included in the model were age, sex, presence of comorbidity, Eastern Cooperative Oncology Group performance status (ECOG PS), tumour site, clinical stage, tumour grade and travel time. The Statistical Package for the Social Sciences software (version 23, IBM Corporation) was used to perform all statistical analyses. A *p*-value of less than 0.05 was considered statistically significant.

## Results

A total of 263 patients met our eligibility criteria and were included in the analysis. Two-thirds of patients (*n* = 178, 67.7%) belonged to Group A, i.e., commuted from their home for treatment. The demographic characteristic of these patients is presented in Table 1. Group A patients traveled 22.0 km (9.4–42 km), spending 48 (24–76.5) minutes one way to reach the cancer center. The symptom duration was 3 (2–6) months, which did not correlate with travel time (correlation coefficient *ρ* = 0.018, *p* = 0.8). Travel time was also similar between patients presenting with advanced disease versus those with early-stage disease (51 versus 48 minutes, *p* = 0.7). Travel time also did not correlate with time to treatment initiation (correlation coefficient *ρ* = −0.037, *p* = 0.6).

Group B patients, who rented an apartment near the cancer center for treatment, comprised 85 patients (32.3%) whose demographic characteristics are presented in Table 1 and are similar to those of patients in Group A. If not for the nearby apartment, Group B patients would have had to travel 75 (65–89.5) km from home to reach the cancer center, spending 130 (110–156) minutes one way. Symptom duration and time to initiation of treatment did not correlate with these putative travel times. Group B patients stayed near the hospital until the completion of their treatment protocols, which was a median duration of 13.4 (IQR, 9.9 to 15.7) months.

### Correlation with receipt of treatment

Among Group A patients, 7.3% did not receive any treatment (surgery, chemotherapy or radiotherapy) – this proportion was higher among patients with the highest travel burden (top quartile of travel time, Q4) versus patients in the lower three quartiles (14% versus 5%). Patients who did not receive treatment also had a higher mortality risk (unadjusted HR: 2.9, 95% CI: 1.1 to 7.4; *p* = 0.02, Log Rank test).

### Correlation with survival outcomes

**Figure 1.**
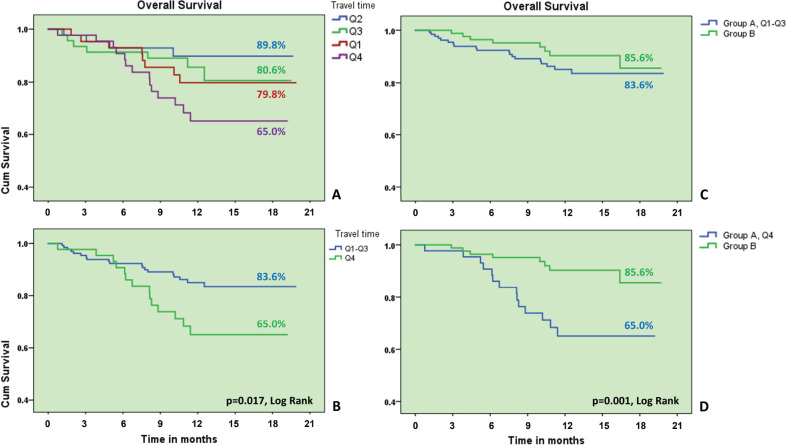
Survival analysis of patients with CRC stratified by travel time (18-month OS labeled adjacent to the respective curves). Patients in the top quartile of travel time (Q4) have poorer survival than patients in the other three quartiles (Q1, Q2, and Q3), both individually (panel A) and combined (panel B). Patients in travel time quartiles Q1–Q3 have similar survival to patients in group B (panel C). However, patients in the top quartile of travel time (Q4) have poorer survival than patients in group B (panel D).

Among patients in Group A, death was observed in 18.5% patients with an 18-month OS rate of 78.9% (95% CI 72.2% to 85.6%). The unadjusted 18-month OS among patients with the highest travel burden (top quartile of travel time, Q4) was 65.0% (95% CI 49.9% to 80.1%) compared to 83.6% (95% CI 76.5% to 90.7%) among patients in the other three quartiles (Q1–Q3) (Figure 1). After adjusting for disease parameters such as metastatic disease, tumour grade, receipt of appropriate treatment and other variables, patients living farthest from the hospital had a significantly poorer survival (adjusted HR 2.5, 95% CI 1.2 to 5.2, *p* = 0.02), independent of other disease parameters (Table 2).

The 18-month OS rate among patients in Group B was higher (85.6%, 95% CI 74.4% to 96.8% versus 78.9%, 95% CI 72.2% to 85.6%) than that of patients in Group A (HR: 2.1, 95% CI 1.0 to 4.5, *p* = 0.056). Group A patients with less travel burden (Q1–Q3) had similar OS to patients in Group B, but the OS of Group A patients with the highest travel burden (Q4) was significantly worse than that of patients in Group B (18-month OS of 65.0% versus 85.6%; HR: 3.7, 95% CI 1.6 to 8.9; *p* = 0.003) (Figure 1).

## Discussion

In this analysis of travel burden among patients with CRC in an RLS of Kashmir, India, we identified that one-third of patients were forced to leave their homes and had to rent an apartment near the cancer center to avoid travel burden for completing their cancer treatment. In addition, for patients who commuted from their homes, patients who lived farthest from the cancer center were at a higher risk of not receiving appropriate treatment and also had worse survival outcomes. Therefore, our study highlights travel burden as an important factor to consider in treatment decisions. These findings are important for cancer policy decisions in RLS.

The magnitude of travel burden in our study was less than the values reported from other RLS in previous studies. In neighboring country Nepal, patients commute more than 24 hours one way to reach cancer centers [5]. In a previous study from the Philippines, patients needing radiotherapy traveled an average of 101 km [6]. Similarly, in Uganda, patients with cervical cancer traveled up to 14 hours to receive treatment [7]. One reason for this lesser travel burden could be that patients with a very long commute time did not even come for treatment at our cancer center. Besides, patients who live in very remote parts of Kashmir may also not be very educated and financially stable. Thus, they could have never encountered cancer care services. Similarly, patients from some other places that are far away from our cancer center could have traveled to other neighboring states or even to major metropolitan cities for cancer care, depending on their economic status. Indeed, our sample is highly selected in that our patients are mostly members of middle-class families who live within a reasonable distance from the cancer center but cannot travel outside of the region seeking better care.

Studies exploring the relationship between travel burden and survival for patients with CRC are sparse. A heavy travel burden was associated with a higher 90-day and 5-year mortality rate in patients undergoing surgery for gastrointestinal malignancies, including CRCs [8]. We noted similar results, as patients having the highest travel time (top quartile) were at a 2.5-fold higher hazard of death as compared to other patients traveling to the cancer care center. Also, these patients were approximately at a four-fold higher hazard of death as compared to patients who lived even further from the hospital but took up accommodation near the hospital to compare treatment. Even in high-income countries like the U. S., patients with travel distance >50 miles were at a higher risk of presenting with metastatic disease, but the association with mortality was not studied [9]. Similarly, some other studies have shown that people living in rural areas and facing travel burden have less adherence to colonoscopy surveillance [10]. However, no prior studies to our knowledge have specifically shown relationships between travel burden and mortality among patients with CRC.

Reasons for poorer outcomes in patients with higher travel burden are multidimensional. Although one reason for this is the correlation of travel burden with advanced-stage presentation due to diagnostic delays [9], this was not the case in our study. However, travel burden can also adversely affect treatment selection and compliance, resulting in attrition, compromised quality of care and poorer outcomes [11, 12]. Studies have shown that distance from the patient’s residence to the hospital is proportional to an increase in length of stay in the hospital, even in elective colorectal surgeries [13] and correlates with readmission rates after most colorectal surgeries [11]. Increased travel burden also decreases the likelihood of receiving appropriate treatment, including adjuvant chemotherapy [14, 15] and palliative chemotherapy [16]. These results are highly consistent with our own. In our cohort, the lack of receipt of treatment was significantly higher among patients with the highest travel burden, and this also led to higher mortality rates.

Additionally, although we did not study this specifically, other studies have also shown that travel burden has a significantly negative impact on the different dimensions of quality of life for patients with cancer [17–19]. Besides fatigue and emotional exhaustion highlighted in these studies, travel burden and financial toxicity are also intricately related. Indeed, travel burden has been included as an important component of the financial toxicity of cancer care in low-/middle-income countries [20]. Even in high-income countries, travel costs constitute the highest proportion of non-medical costs for patients with cancer [21]. Some previous studies have also shown the association of financial toxicity with treatment adherence and mortality outcomes, showing overlap between the travel and financial burdens [22].

Our cohort of Group B patients, who rented a place near the hospital to receive cancer therapy, is unique and deserves special mention. They had better survival outcomes; however, whether that survival benefit is solely the result of living near the cancer center is unknown. Being near would mean easy access to care, which can have a positive impact on outcomes; however, these patients may also be systematically different from Group A patients in terms of socioeconomic parameters. These patients were educated and motivated enough to prioritise cancer treatment, and also rich enough to afford renting a place near the cancer center. These factors can independently lead to improved survival rates. Thus, it is not possible to ascribe improved survival directly to renting nearby accommodation.

Several limitations must be considered when interpreting our results. First, as discussed above, our patient group may not necessarily be reflective of other RLS. Other studies from other RLS should consolidate our findings. In addition, given the quality of our database, we could not control for all other factors, such as socioeconomic factors, hospitalisation rates and so on, that could affect the outcomes. In addition, as highlighted above, there could be substantial overlaps between travel burden and financial toxicity. We are currently running prospective studies to collect more information on financial toxicity and its impact on outcomes among patients with cancer in Kashmir. More importantly, patients with the most severe time burden, such as those living in the remotest parts of Kashmir, are probably excluded from our analysis because they did not even present for cancer treatment. Thus, our results are most likely an underestimate of the magnitude of the problem.

## Conclusion

Our study demonstrates that patients with CRC who are residing farthest from the comprehensive cancer care centers are at a higher risk of poorer outcomes. Health systems should ensure that patients have access to cancer treatments within a reasonable distance of their residence to optimise treatment and follow-up. Focused strategies need to be devised to mitigate the addressable challenge of travel burden in patients with cancer.

## Conflicts of interest

No authors have any conflicts of interest to report related to the manuscript. Unrelated to the manuscript, BG has received consulting fees from Vivio Health.

## Funding

No funding was received for performing the study or preparing the manuscript. Publication fees were covered by funds from BG Lab, Queen’s University.

## Disclosures

None reported.

## Ethical approval

This study was approved by the Institutional Ethics Committee of SKIMS Srinagar vide no. SIMS 131/IEC-SKIMS/2023-181.

## Author contributions

**SZB:** Inception of idea/study design, arrangement of resources, drafted and edited the manuscript, patient management and follow-up, data acquisition/analysis and review of literature.

**MTR, SZA, LS, MA:** Arrangement of resources, edited the manuscript, patient management and follow-up and data acquisition.

**AZB:** Inception of idea/study design, drafted and edited the manuscript, data analysis and review of literature.

**BG:** Inception of idea/study design, drafted and edited the manuscript, review of literature and overall supervision of manuscript preparation.

All authors approve the final version of the manuscript.

## Figures and Tables

**Table 1.** Baseline char acteristics of patients included in our study.

Parameter	Group A (n = 178)	Group B (n = 85)
Age (years)	54.8 ± 14.9	54.6 ± 13.7
Female sex	43.8% (*n* = 78)	40.0% (*n* = 34)
Presence of comorbidity	43.8% (*n* = 78)	35.3% (*n* = 30)
ECOG PS >1	15.2% (*n* = 27)	14.1% (*n* = 12)
Distance (km)	22.0 (9.4, 42.0)	75.0 (65.0, 89.5)
Travel time (mins)	48.0 (24.0, 76.5)	130.0 (110.0, 156.0) [putative]
Symptom duration (months)	3.0 (2.0, 6.0)	3.0 (1.5, 8.0)
Baseline CEA levels (ng/mL)	4.44 (2.19, 11.00) (*n* = 135)	3.49 (2.10, 12.30) (*n* = 63)
Clinical stage – localized Locoregional Metastatic	9.6% (*n* = 17) 71.3% (*n* = 127) 19.1% (*n* = 34)	14.1% (*n* = 12) 67.1% (*n* = 57) 17.6% (*n* = 15)
Site – right colon Left colon (including rectum)	20.2% (*n* = 36) 73.6% (*n* = 131)	20.0% (*n* = 17) 74.1% (*n* = 63)
Tumour grade – 1 2 3	19.1% (*n* = 34) 60.1% (*n* = 107) 20.8% (*n* = 37)	20.0% (*n* = 17) 60.0% (*n* = 51) 10.6% (*n* = 9)

**Table 2.** Parameters predicting outcomes in patients with CRC included in our study (data expressed as HR (95% CI), p-value).

Parameter	Group A		Group B	
	**Univariate analysis**	**Multivariate analysis**	**Univariate analysis**	**Multivariate analysis**
1. Age >50 years	1.6 (0.8 to 3.5), 0.2		1.3 (0.3 to 6.4), 0.8	
2. Female sex	0.8 (0.4 to 1.5), 0.4		0.5 (0.1 to 2.3), 0.3	
3. Presence of comorbidity	1.1 (0.5 to 2.1), 0.8		0.0 (0.0 to 8.0), 0.2	
4. ECOG PS >1	3.6 (1.7 to 7.5), 0.001	3.4 (1.4 to 7.9), 0.005	0.8 (0.1 to 6.7), 0.9	
5. Left versus right colon	1.3 (0.5 to 3.5), 0.6		1.6 (0.12 to 13.4), 0.7	
6. Metastatic disease	8.1 (4.0 to 16.2), <0.0001	4.9 (2.3 to 10.6), <0.0001	10.5 (2.4 to 45.5), 0.002	8.4 (1.7 to 42.3), 0.01
7. G3 tumor grade	8.2 (4.1 to 16.6), <0.0001	5.7 (2.7 to 12.2), <0.0001	0.0 (0.0 to >100), 0.6	
8. Travel time top quartile	2.3 (1.2 to 4.6), 0.02	2.5 (1.2 to 5.2), 0.02	-	
9. Receipt of treatment	2.9 (1.1 to 7.4), 0.02		-	
